# Role of Protease-Inhibitors in Ocular Diseases

**DOI:** 10.3390/molecules191220557

**Published:** 2014-12-08

**Authors:** Nicola Pescosolido, Andrea Barbato, Antonia Pascarella, Rossella Giannotti, Martina Genzano, Marcella Nebbioso

**Affiliations:** 1 Department of Cardiovascular, Respiratory, Nephrologic, Anesthesiologic and Geriatric Sciences, Policlinico Umberto I, Faculty of Medicine and Odontology, Sapienza University of Rome, piazzale Aldo Moro 5, Rome 00185, Italy; 2 Department of Sense Organs, Ocular Electrophysiology Center, Policlinico Umberto I, Faculty of Medicine and Odontology, Sapienza University of Rome, piazzale Aldo Moro 5, Rome 00185, Italy; 3 Department of Biology and Biotechnology Charles Darwin, Sapienza University of Rome, piazzale Aldo Moro 5, Rome 00185, Italy

**Keywords:** age-related macular degeneration, endophthalmitis, keratitis, keratoconus, neurodegenerative disorders, optic neuritis, protease-inhibitors, retinal ganglion cells, Sorsby dystrophy

## Abstract

It has been demonstrated that the balance between proteases and protease-inhibitors system plays a key role in maintaining cellular and tissue homeostasis. Indeed, its alteration has been involved in many ocular and systemic diseases. In particular, research has focused on keratoconus, corneal wounds and ulcers, keratitis, endophthalmitis, age-related macular degeneration, Sorsby fundus dystrophy, loss of nerve cells and photoreceptors during optic neuritis both *in vivo* and *in vitro* models. Protease-inhibitors have been extensively studied, rather than proteases, because they may represent a therapeutic approach for some ocular diseases. The protease-inhibitors mainly involved in the onset of the above-mentioned ocular pathologies are: α2-macroglobulin, α1-proteinase inhibitor (α1-PI), metalloproteinase inhibitor (TIMP), maspin, SERPINA3K, SERPINB13, secretory leukocyte protease inhibitor (SLPI), and calpeptin. This review is focused on the several characteristics of dysregulation of this system and, particularly, on a possible role of proteases and protease-inhibitors in molecular remodeling that may lead to some ocular diseases. Recently, researchers have even hypothesized a possible therapeutic effect of the protease-inhibitors in the treatment of injured eye in animal models.

## 1. Introduction

Proteases are widespread enzymes in all plants, animals and micro-organisms. For more than one hundred years, studies have been carried out on their well-known property to catalyze hydrolysis of certain peptides in target proteins. Later, more evidence of their importance in many biological processes has been object of studies in all living organisms. Thus, proteases regulate the fate, localization and activity of many substrates, create new bioactive molecules, contribute to process cellular information, transduce and amplify molecular signals and modulate protein-protein interactions [1,2].

Despite that this group of enzymes being vital to their host cells, they may be also potentially damaging when over-expressed or present in higher concentrations; e.g., they activate in cancer, neurodegenerative disorders, inflammation and cardiovascular diseases. For this reason the function of this group of enzymes need to be strictly regulated and supervised. 

**Table 1:** Molecules most commonly involved in maintaining cellular and tissue homeostasis.

Ocular Disorders and Site	Proteases and Protease-Inhibitors Mainly Involved in Ocular Diseases		
Degenerative Disorder: keratoconus and keratoglobus	α2-macroglobulin↓ α1-proteinase inhinitor (α1-PI)↓	Cathepsin B and G↑ Matrix Metalloproteinase-1,2,3 (MMP-1,2,3)↑	Acid phosphatase↑ Catalase↑
Keratitis: angiogenesis, tumor, inflammation, oxidative stress, and fibrosis activity	Serpins↑: ovalbumin, α1-antitrypsin, plasminogen activator inhibitor, maspin, pigment epithelial derived factor (PEDF), SERPINA3K	Cathepsin↑ MMP-8↑	Calpain inhibitor↑ Catalase↑
Iris and ciliary body	Secretory leukocyte protease inhibitor (SLPI)↑	MMPs↑	Calpain inhibitor↑
Endophthalmitis, keratitis, vitreous and retina disorders	SLPI↑	MMPs↑	Calpain inhibitor↑
Retina: angiogenesis, tumor, inflammation, oxidative stress, and fibrosis activity	SERPINA3K↑	Metalloproteinase inhibitor-3 (TIMP-3)↓ [age-related macular degeneration, aging, Sorsby dystrophy]	Calpain inhibitor↑
Retina/optic nerve: damage to the retinal ganglion cells (RGC) in glaucoma, multiple sclerosis (MS), retinitis pigmentosa, diabetes, *etc.*	Caspase 3 inhibitor↑ [apoptosis of RGC and damage to optic nerve fibers]	MMPs↑	Calpain inhibitor↑ [apoptosis of RGC, damage to optic nerve fibers and photoreceptors]

The most important control system is the protease-inhibitors (PIs) one (Table 1) represented by proteins that form less active or fully inactive complexes with their cognate enzymes [2]. For a few PIs, other functions than just blocking proteases have also been found, such as growth factors activity, clearance receptors or carcinogenesis promotion. PIs have been divided into families and sub-families according to their amino-acid sequences and the relationship between protein foldings of the inhibitory domain in each member of the class [2,3].

Serpins, cysteines and metallocarboxypeptidase inhibitors are the most common families expressed in human beings [4]. 

In this review we evaluate some characteristics of the proteases and protease-inhibitors and their involvement in ocular pathological conditions, especially corneal and retinal degenerative disorders, enlightening the evidence of their role in molecular remodeling. Recently, researchers have even hypothesized a possible therapeutic effect of protease-inhibitors in topical and systemic treatment of neovascularization and inflammation in animal models, suggesting that they also may play a neuroprotective and antimicrobial role.

## 2. Protease-Inhibitors and Eye Diseases

### 2.1. Keratoconus

**Figure 1:**
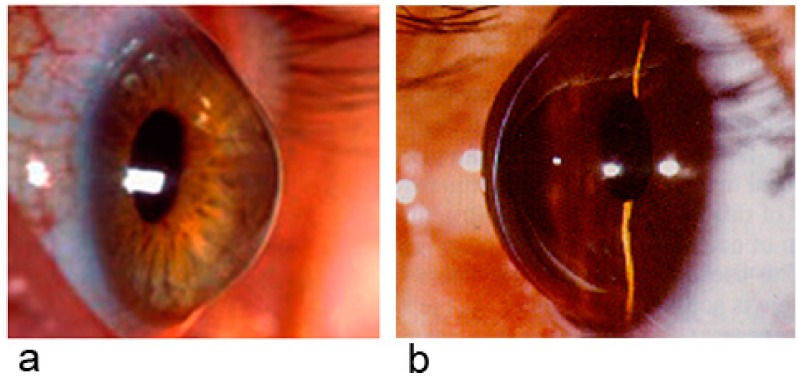
*Keratoconus*. Left panel (**a**). Progressive and non-inflammatory disorder of the eye characterized by ectasia of the central and/or inferior portion of the cornea that induces irregular astigmatism and often myopia, resulting in mild to marked visual impairment. *Keratoglobus.* Right panel (**b**). Histopathologic and immunohistochemical abnormal features are similar in keratoglobus and keratoconus. In addition, the cornea in keratoglobus is diffusely thinned, often more markedly in the peripheral cornea, whereas in keratoconus the thinning is most prominent in the central cornea.

Keratoconus is a progressive non-inflammatory disorder of the eye characterized by ectasia of the central and/or inferior portion of the cornea that induces irregular astigmatism and often myopia, resulting in mild to marked visual impairment [5,6] (Figure 1a). Progressive dissolution of Bowman’s layer can be observed in keratoconus eyes, besides a ring from yellow-brown to olive-green pigmentation known as “Fleischer’s ring” and other lesions in the central portion of the cornea. Moreover, cellular and structural changes in the cornea adversely affect its integrity and lead to the bulging and scarring characteristics of the disorder. Within any individual keratoconus cornea, it is possible to find areas of degenerative thinning at the same time as areas undergoing wound healing. Keratoconus has been associated with eye rubbing, atopy and contact lens wear [5,6]. An estimate of the prevalence for keratoconus is 1 in 2000 individuals, but it is diagnosed 300 times more in patients with Down’s syndrome [7,8,9]. 

The etiopathogenesis of the disease is still not completely clear, although some genetic components may be involved. Several biochemical analyses showed that corneas obtained from patients with keratoconus have significantly less total proteins per milligram of dry weight than those from controls. The protein synthesis in some keratoconus corneal cultures, however, was found to be normal. These results have led to the theory that degradation of macromolecules, including proteases and protease-inhibitors, may be one of the mechanisms involved in the genesis of keratoconus. 

### 2.2. Keratoconus and α2-Macroglobulin 

Moreover, α2-macroglobulin is a high-molecular weight (718 kd) homotetrameric glycoprotein involved in protein degradation as well as protection and regulation of cytokine molecules [8,9,10]. In 1994 Sawaguchi *et al.* measured the levels of α2-macroglobulin from 46 patients with clinical features typical of keratoconus compared to corneal buttons from 50 normal human eyes [11]. The result of this study, in which a Western blot assay was performed after immunoperoxidase technique, showed that the staining intensity of α2-macroglobulin in epithelial cells and in the thinnest keratoconus areas was markedly reduced compared to non-pathological corneas. However, the exact mechanism underlying the α2-macroglobulin aberration in keratoconus is unknown. The abnormality could be related to a lower biosynthesis or an increased degradation of the inhibitor, or to some changes in teardrops or aqueous humor. It is known that corneal cells can synthesize α2-macroglobulin and the inhibitor has been demonstrated in tears and aqueous humor as well [12,13].

### 2.3. Keratoconus and Dysregulation

In addition, Twining *et al.* in 1994 demonstrated that αl-antitripsin (proteinase inhibitor) is also synthesized and released by human corneal epithelial cells. Their results indicated that the cornea has the ability to locally control degradation through synthesis of the inhibitor without total dependence on a supply of the inhibitor itself from vascular system [14]. Again, the dysregulation between degradation enzymes and their inhibitors has been genetically proved too, by polymerase chain reaction (PCR), especially for cathepsin G, acid phosphatase and α1-proteinase inhibitor (α1-PI). Cathepsin G is a neutral serine protease, known for its capability to degrade proteoglycans and collagen of articular cartilages and enhance elastase activity *in vitro*, while αl-PI is one of the major protease-inhibitors in plasma and member of the serpin family [14]. 

Whitelock *et al.* examined the gene expression of these enzymes in keratoconus corneas. In particular, the acid phosphatase and cathepsin G mRNA levels were found to be increased. Instead, α1-PI was found to be markedly reduced, especially in the epithelial layer of keratoconus corneas [15].

Besides using PCR, Zhou *et al.* have screened a spectrum of degradation enzymes and inhibitors by immunohistochemical staining, Western blot analysis and immunoenzymatic assays. They demonstrated that the level and activity of cathepsins B and G were increased in corneas with keratoconus. Cathepsin B is a cysteine protease with degradation activities *versus* major extracellular matrix (ECM) components of the corneal stroma. The expressions of various other enzymes, including urokinase, matrix metalloproteinase (MMPs), and protease-inhibitors, (plasminogen activator inhibitor-1, α1-antichymotrypsin, α2-antiplasmin, TIMP-1, and TIMP-2), were unaltered in keratoconus [16]. The results obtained from immunohistochemical experiments corroborated their data that the levels of MMPs are not modified in keratoconus, but *in situ* zymography demonstrated that basal levels of net gelatin- and casein-digesting activities, present in healthy human corneas, were increased in keratoconus. Gelatin and casein are the best substrates for gelatinases A (MMP-2) and B (MMP-9), and stromelysin (MMP-3). They can, however, also serve as substrates for other proteinases [16,17,18]. 

To determine whether the activities observed were caused by MMPs or other classes of proteinases, Zhou *et al.* used specific inhibitors for the four classes of proteinases (aspartic, serine, cysteine and metallo). Their results indicated that in healthy controls and keratoconus specimens, the net gelatinolytic and caseinolytic activities were related mostly to serine and cysteine proteinases, not to aspartic proteinases, gelatinases A and B, or stromelysin [16,17].

The up- or down-regulation of the enzyme and inhibitor genes were noted at both mRNA levels. Since multiple gene involvement and the possibility of a coordinated gene regulation mechanism were supposed, several transcription factors were examined. Among them, Sp1 was found specifically up-regulated in keratoconus corneas [17,18].

In addition, Shen *et al.* have demonstrated that up-regulation of Sp1 suppresses the promoter activity of the human α1-PI gene in corneal cells [19], while the up- or down-regulation of lysosomal acid phosphatase, cathepsin B, and α2-macroglobulin observed in keratoconus-affected corneas were not mediated by Sp1. It is possible that in corneal epithelium, the Sp1/α1-PI abnormality contributes to degradation and breaks of the epithelial basement membrane zone, resulting in the earliest pathologic features. Furthermore, over-expression of Sp1 not only down-regulate the α1-PI gene, but also represses corneal cells proliferation [18,19]. As for the Sp1 over-expression, the increased Krüppel-like factor 6 (KLF6) may suppress the expression of the α1-PI gene in keratoconus. KLF6 is a member of the family of Krüppel factors. This family is closely related to the Sp1 transcription factor family. Western blot analyses revealed that KLF6 level in epithelial cells derived from keratoconus corneas was higher than that from normal corneas. Direct physical interaction between KLF6 and Sp1 has been documented by co-immunoprecipitation [20]. 

The abnormalities in cathepsins B and G, α1-proteinase inhibitor, α2-macroglobulin and Sp1 identified in keratoconus corneas were not evident in the conjunctiva, thus keratoconus appears to be a disease localized to the cornea [21].

### 2.4. Oxidative Stress in Corneal Degenerative Disorder

In a recent study [22] keratoconus corneas showed evidence of oxidative damage. 

Reactive oxygen species (ROS) accumulation can result in altered enzyme activities, inhibition of DNA/RNA/protein synthesis, and other damaging events. The healthy cornea is rich in antioxidant enzymes such as superoxide-dismutase (SOD), catalase, glutathione peroxidase, and glutathione reductase, all involved in the removal of free radicals and ROS [22].

Recently, several lines of evidence have emerged suggesting that corneal components of protection against oxidative damage inducible nitric oxide synthase (iNOS) and glutathione S-transferase (GST) were altered in keratoconus corneas, while extracellular forms of SOD and aldehyde dehydrogenase (ALDH)-3A1 have been reported to be reduced [22,23,24].

Corneal ALDH is the major soluble protein of the mammalian cornea and it has an important role in detoxification of cytotoxic aldehydes that are generated in cells after membrane lipid peroxidation. Aldehydes disrupt the membranes of lysosomes and cells, releasing lysosomal proteolytic enzymes, including cathepsins [25,26].

In 2005, Kenny *et al.* evidenced an important role of the oxidative stress in corneal degenerative disorder [27]. The enzymes examined were catalase, cathepsin, MMPs, SOD, glutathione reductase, GST and ALDH-3A1. Their results showed that keratoconus corneas have elevated levels of cathepsins, which can stimulate hydrogen peroxide production and up-regulate catalase [27].

### 2.5. Keratoglobus and Keratoconus

Moreover, histopathologic and immunohistochemical abnormal features are similar in keratoglobus and keratoconus [28] (Figure 1a,b). However, the cornea in keratoglobus is diffusely thinned, often more markedly in the peripheral cornea, whereas in keratoconus the thinning is most prominent in the central cornea. Keratoglobus has been described as both an acquired and congenital disease. Acquired keratoglobus has been associated with dysthyroid ophthalmopathy, vernal keratoconjunctivitis, and chronic marginal blepharitis [29]. Eye rubbing has been proposed to be the major contributing factor in the latter two entities.

Immunohistochemistry performed on corneas with keratoglobus revealed that the expression of α1-PI and Sp1 was similar to that of keratoconus, and MMP-1, MMP-2, and MMP-3 immunostaining intensity in keratoglobus was significantly higher than that seen in normal controls. In summary, the current results suggest that the stromal thinning in keratoglobus and keratoconus may arise by degradation processes and that the two conditions may share common, as-yet-unclear pathogenic pathways, though extra degradation events may occur in keratoglobus. Besides the keratoconus onset and progression, proteases and protease-inhibitors capability to remodel normal turnover of the ECM suggests a possible role in corneal wound healing [28].

### 2.6. Cornea and Serine Protease Inhibitors

Great interest is currently being expressed for the class of serine protease inhibitors (serpins), especially maspin [30]. In humans, the gene encoding maspin is located on chromosome 18, in a cluster containing genes for other serpins such as α1-antitrypsin, plasminogen activator inhibitor, pigment epithelial derived factor (PEDF), and ovalbumin [30,31,32].

Maspin was classified as a class II tumor suppressor gene in human mammary epithelial cells because its expression, although down-regulated in many metastatic carcinoma cells, is not mutated. Despite its structure resembles the one of a classical inhibitory serpin, the reactive site (or center) loop possesses a non-standard hinge region, that prevents maspin from being able to trap and inhibit serine proteases [30]. In the human cornea, maspin is expressed not only by the epithelial cells, but also by stromal keratocytes and endothelial cells. The corneal stromal cell is the first non-epithelial cell type shown to synthesize maspin. Although maspin is synthesized by corneal keratocytes *in situ* and by primary and first-passage corneal stromal cells in culture, its expression declines to undetectable levels in later passage cells. Later passage cultured stromal cells have protein expression patterns that are similar to those of cells in wounded corneal stroma. Down-regulation of maspin expression in corneal stromal cells is similar to the one in mammary gland epithelial cells after turning into carcinoma [31]. In carcinoma cells, increased cell adhesion to fibronectin results in decreased tumor cell migration. Maspin may similarly regulate corneal stromal cell migration by increasing cell adhesion to fibronectin, raising the possibility that maspin plays a regulatory role in stromal wound healing. A possible sequence of events in wounded stroma may be that when conversion of cells to fibroblasts takes place, it results in down-regulation of maspin expression, low maspin levels in the cornea, and therefore creation of a permissive environment for their migration [32,33]. During the modification of stromal keratocytes into corneal wound healing cells, hypermethylation and histone H3 dimethylation are involved in the down-regulation of maspin gene [34].

### 2.7. Angiogenesis, Inflammation, Oxidative Stress, and Serine Protease Inhibitors

Maspin may also have other functions in corneal stroma, besides its famous antiangiogenic properties which contributes to the avascolarity of the cornea and transparency as well. Several serpin family members hold tumor suppressor functions including angiogenesis inhibition [35,36]. In a rat corneal assay of neovascularization, SERPINA3K (Hurpin/Headpin) protein blocked *in vivo* angiogenesis mediated by interleukin 8 (IL-8) and vascular endothelial growth factor (VEGF) [37].

In addition Liu *et al.* evaluated the therapeutic effects of SERPINA3K on neovascularization and inflammation in a rat alkali burn cornea model that is commonly used to study corneal wounding. Topical treatment of the injured rat cornea with SERPINA3K (20 μg/eye/day) for 7 days significantly decreased the neovascular area. Furthermore, SERPINA3K enhanced the recovery of corneal epithelium after the alkali injury. In particular, SERPINA3K down-regulated the expression of the pro-angiogenic and pro-inflammatory factors, VEGF and Tumor Necrosis Factor-α (TNF-α) and up-regulated the expression of PEDF and epidermal growth factor receptor [38].

Hence, SERPINA3K was first identified as a specific inhibitor of tissue kallikrein, but later studies have reported that it has effects of anti-angiogenesis, anti-inflammation, anti-fibrosis, and anti-oxidative stress at retinal level [37,38,39,40].

### 2.8. Neurodegenerative Disorder in Retina and Optic Nerve

Non-regulation of neural apoptosis and angiogenesis is involved in various diseases such as neurodegenerative disorders, neuronal trauma, glaucoma, and inflammation in the optic nerve with subsequent damage to the retinal ganglion cells (RGC) [39,40,41,42,43,44,45,46]. 

**Figure 2:**
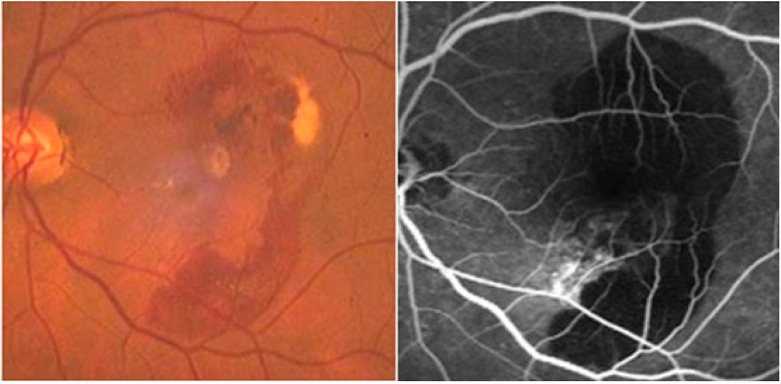
Age-related macular degeneration (AMD) and fluorescein angiography.

**Figure 3:**
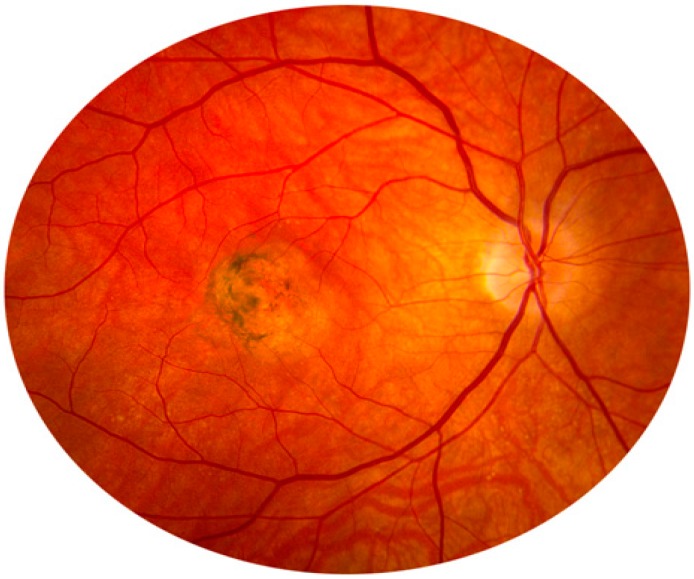
Sorsby fundus dystrophy.

It has previously been suggested that TIMP-3 is a senescence-related protein. In fact, some studies have shown elevations in TIMP-3 protein levels in Bruch’s membrane of patients with age-related macular degeneration (AMD) (Figure 2) and during normal aging. More than 98% of subjects over 50 years of age were found to have moderate to strong ECM TIMP-3 staining in Bruch’s membrane [41]. Similarly, the manifestations of Sorsby fundus dystrophy (SFD), a dominant inherited condition, are characterized by the development of choroidal neovascular membranes, subretinal hemorrhages, lipid-rich extracellular deposits in Bruch’s membrane and disciform macular degeneration (Figure 3). This disease is similar to AMD, but usually occurs from the third to fourth decade of life. It has been established that SFD is caused by some mutations in the tissue TIMP-3 gene [42]. The researchers hypothesized that TIMP-3 shows some kind of antiangiogenic activity and could be required to maintain a controlled degradation of the ECM around the blood vessels in the retina. Therefore, a mutation in this protein may disrupt the balance and lead to excessive degradation of the ECM, resulting in choroidal and retinal neovascularization. 

Some authors stated that calpains and CPP32-like caspases are implicated in the pathologic cascade by proteolysis and secondary axotomy and are the major mediators of apoptosis in RGC after damage to the optic nerve. The inhibition of the proteases in experimental models of neuronal trauma or demyelinating disease increases the number of surviving neurons after axonal lesion [43,44,45,46]. These results provide evidences for the activation of proteases as a general mechanism of neuronal apoptosis. Consequently, inhibition of proteases might be a very promising strategy in the treatment of various forms of brain injury, including trauma, optic neuropathies, and glaucoma. Likewise, some authors describe that a high quantity of fat and hyperglycemia results to start a sequence of calpain activation and oxidative stress associated with neuro-degenerative changes in diabetic retinopathy and significant increases of RGC and axonal degeneration 3 weeks from the experiment. Therefore, antioxidants and calpain inhibitors offer important opportunities for future neuroprotective treatment against RGC death in various metabolic stress-induced diseases including diabetic retinopathy [45,46,47,48].

Among antioxidant molecules, alpha-lipoic acid (ALA) and SOD could counteract neurodegenerative deterioration to the retina and optic nerve in animal models of diabetic retinopathy, senescence and glaucoma. In a recent study carried out by our research group, oral administration of ALA and SOD in aged rats improved the condition of ocular nerve tissue, otherwise subject to the normal ageing process (*i.e.*, oxidative stress). It was shown that ALA and SOD are able to negatively modulate iNOS enzyme and induce a down-regulation in the expression of caspase-3, a marker for the final phase of the apoptotic cascade [47,49]. Furthermore, the lipid peroxidation assay confirmed that, following administration, there is less damage to membranes, thus protecting retinal cells and optic nerve fibers from tissue death [49]. Perhaps ALA and SOD might reduce the activity of proteases because they could directly inhibit their action or increase the effect of their inhibitors.

## 3. Eye Infections and Inflammations by Protease Inhibitors

Persson *et al.* examined by immunohistochemistry the localization of the extralysosomal calpain-proteases in the eye of the rabbit [50]. Immunoreactivity against the calpains was observed in the epithelial cells of the cornea, iris and ciliary body. The sclera and choroid layers showed a weak immunoreactivity. Besides, the various retina layers, pigment epithelium, plexiform layers, Müller cells, receptor cells, RGC, and nerve fiber layer, were heavily labeled. The authors concluded that the results could be compatible with a role of the calpains in the secretory/phagocytic process and as modulators of the cytoskeleton in cell processes [50,51].

What has been declared before about non-infectious corneal wounds may also work for corneal ulceration, in fact, combined antibiotic therapy with protease-inhibitors can speed corneal healing, because MMPs are involved in keratitis, corneal ulceration, and endophthalmitis as well: these pathologies may cause blindness and persist for a lifetime [40,50,51,52,53,54]. Immunohistochemical studies performed on murine model confirmed high levels of MMP-8, IL-1, IL-6, TNF-α, and SLPI (secretory leukocyte protease-inhibitor) in eyes with *S. aureus* keratitis and epithelial defects, in opposition to undetectable SLPI expression in normal control corneas. SLPI is composed of two domains: a protease-inhibitor at the *C*-terminal domain and an antimicrobial at the *N*-terminal one. Reviglio *et al.* demonstrated that SLPI is not synthesized in ocular tissues under normal physiologic conditions, but it may be present in order to promote the early eradication of invasive micro-organisms and protect the inflamed cornea, vitreous, and retina tissues of rat eyes with *S. Aureus* endophthalmitis against proteolytic destruction mediated by inflammatory cells [53,54].

Smith *et al.* showed that, in animal models for multiple sclerosis (MS) of experimental autoimmune encephalomyelitis (EAE), the expression of apoptotic and inflammatory proteins was determined by Western blotting after daily intraperitoneal injections of calpeptin. The last one is a calpain-inhibitor, a molecules widely distributed all over the eyeball [55]. It was demonstrated that calpeptin down-regulates the expression of proapoptotic proteins and proinflammatory molecule nuclear factor-κB in the retina of Lewis rats with acute EAE. A moderate dose of calpeptin dramatically reduced the apoptotic loss of RGC. For this reason, calpain inhibition might be a useful supplement to immunomodulatory therapies such as corticosteroids in optic neuritis, due to its neuroprotective effect on RGC [55]. In addition, other authors stated that the calpeptin treatment could maintain the thickness of retinal nerve fibers layer in MS and glaucoma too, thus promoting the preservation of optic nerve axons and as a consequence protect of the vision [55,56]. 

Furthermore, calpain activation is also involved in photoreceptor cell death, and calpain inhibition restores photoreceptor cell autophagy and consequently reduces photoreceptor cell death. This hypothesis has been verified in mice treated with intraperitoneal injection of *N*-methyl-*N*-nitrosourea that induces photoreceptor cell death by apoptosis and is a reliable animal model for human retinitis pigmentosa. Calpain inhibitor (SNJ-1945) ameliorated photoreceptor cell death by blocking calpain activation and restoring basal autophagy [57].

## 4. Conclusions

As a result, multiple factors are involved in the above-mentioned ocular disorders and evidence of the new direction of study could be fundamental for the eye treatment. In the present study we speculate that, in the near future, the regulation of proteases and their inhibitors could represent a therapeutic approach in ocular diseases characterized by elevated proteases activity. In fact, modulation of apoptosis provides opportunities to a new therapeutic strategy for many disorders in which the apoptotic process contributes to the progression of the disease itself. 
